# Evidence for a second class of *S*-adenosylmethionine riboswitches and other regulatory RNA motifs in alpha-proteobacteria

**DOI:** 10.1186/gb-2005-6-8-r70

**Published:** 2005-08-01

**Authors:** Keith A Corbino, Jeffrey E Barrick, Jinsoo Lim, Rüdiger Welz, Brian J Tucker, Izabela Puskarz, Maumita Mandal, Noam D Rudnick, Ronald R Breaker

**Affiliations:** 1 Department of Molecular, Cellular and Developmental Biology, Yale University, P.O. Box 208103, New Haven, CT 06520-8103, USA; 2 Department of Molecular Biophysics and Biochemistry, Yale University, P.O. Box 208103, New Haven, CT 06520-8103, USA; 3 Department of Chemistry, Yale University, P.O. Box 208103, New Haven, CT 06520-8103, USA; 4 Department of Physics, University of California, Berkeley, CA 94720-7200, USA

## Abstract

**Background:**

Riboswitches are RNA elements in the 5' untranslated leaders of bacterial mRNAs that directly sense the levels of specific metabolites with a structurally conserved aptamer domain to regulate expression of downstream genes. Riboswitches are most common in the genomes of low GC Gram-positive bacteria (for example, *Bacillus subtilis *contains examples of all known riboswitches), and some riboswitch classes seem to be restricted to this group.

**Results:**

We used comparative sequence analysis and structural probing to identify five RNA elements (*serC*, *speF*, *suhB*, *ybhL*, and *metA*) that reside in the intergenic regions of *Agrobacterium tumefaciens *and many other α-proteobacteria. One of these, the *metA *motif, is found upstream of methionine biosynthesis genes and binds *S*-adenosylmethionine (SAM). This natural aptamer most likely functions as a SAM riboswitch (SAM-II) with a consensus sequence and structure that is distinct from the class of SAM riboswitches (SAM-I) predominantly found in Gram-positive bacteria. The minimal functional SAM-II aptamer consists of fewer than 70 nucleotides, which form a single stem and a pseudoknot. Despite its simple architecture and lower affinity for SAM, the SAM-II aptamer strongly discriminates against related compounds.

**Conclusion:**

SAM-II is the only metabolite-binding riboswitch class identified so far that is not found in Gram-positive bacteria, and its existence demonstrates that biological systems can use multiple RNA structures to sense a single chemical compound. The two SAM riboswitches might be 'RNA World' relics that were selectively retained in certain bacterial lineages or new motifs that have emerged since the divergence of the major bacterial groups.

## Abstract 2

Comparative sequence analysis and structural probing identified five RNA elements in the intergenic region of *Agrobacterium tumefaciens *and other α-proteobacteria. One of these RNA elements is probably a SAM-II, the only riboswitch class identified so far that is not found in Gram-positive bacteria.

## Background

Riboswitches are structured RNA elements within the noncoding regions of some mRNAs that directly sense metabolites and regulate gene expression [1-4]. Riboswitches are known that respond to a wide range of metabolites including coenzymes [5-8], purines [9,10], amino acids [11,12], and a sugar-phosphate compound [13]. Most riboswitches are found within the 5' untranslated regions of bacterial mRNAs that encode biosynthetic enzymes or metabolite transporters. Ligand binding to the aptamer domain of a riboswitch stabilizes specific structural elements of an adjoining expression platform, which modulates the expression of downstream genes. The two most common types of expression platforms control either the formation of intrinsic transcription terminators that abort mRNA synthesis or the formation of alternate structures that mask ribosome-binding sites to prevent translation initiation.

Riboswitch aptamers have sequence and structural features that are typical of functional RNAs. Each riboswitch class is defined by a core of conserved base-paired elements and consensus nucleotides at specific positions interspersed with variable stems and loops. We have previously used comparative sequence analysis of intergenic regions (IGRs) from 94 microbial genomes to identify conserved RNA motifs residing upstream of functionally related genes in *Bacillus subtilis *that are candidates for new riboswitches [14]. Two of these RNA elements have subsequently proven to be novel riboswitch classes. Candidate RNAs termed *glmS *and *gcvT *function as glucosamine-6-phosphate dependent ribozymes [13] and cooperative glycine riboswitches [12], respectively.

Most riboswitches reported previously are found predominantly in Gram-positive bacteria, and representatives of all classes are present in *B. subtilis*. We speculated that other groups of bacteria might harbor different noncoding RNA domains, some of which could be novel riboswitches. We report here five novel structured RNA elements that were identified by focusing our comparative sequence analysis of IGRs on α-proteobacterial genomes. One of the five newfound motifs from *Agrobacterium tumefaciens*, termed *metA*, appears to function as a riboswitch that senses *S*-adenosylmethionine (SAM). This SAM-II riboswitch class has a consensus sequence and conserved structure that is distinct from the SAM-I riboswitch reported previously [15-18]. Compared with SAM-I aptamers, SAM-II aptamers are smaller and form a simpler secondary structure. However, the SAM-II aptamer exhibits a level of molecular discrimination that is similar to that observed for the SAM-I riboswitch. These findings demonstrate that biological systems use multiple RNA motifs to sense the same chemical compound.

## Results and discussion

### Identification of novel RNA motifs in α-proteobacteria

**Figure 1:**
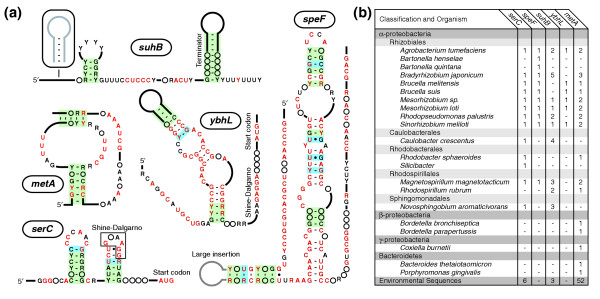
α-Proteobacterial RNA elements. **(a) **Consensus sequences and structures. Red and black positions for each RNA element indicate >95% and >80% conservation of a particular nucleotide, respectively. Purine (R) or pyrimidine (Y) designations are used when a single nucleotide is not >80% conserved. Solid black lines indicate variable regions, and solid grey lines are optional sequence insertions that are not present in all examples of an element. Circles represent single nucleotides whose presence (but not sequence) is conserved. Base pairs supported by strong (both bases in the pair vary) and weak (only one base in the pair varies) sequence covariation in a motif alignment have green and blue shaded backgrounds, respectively. **(b) **Phylogenetic distributions. Element names are chosen based on the proximity that representatives from *A. tumefaciens *have to genes.

We searched α-proteobacterial genomes for new riboswitches and structured regulatory RNA elements by constructing a database of sequence comparisons between IGRs from 116 complete microbial genomes [19] (See also [14] and Materials and methods). We examined alignments and statistics from this database for examples where a conserved sequence motif occurred upstream of genes sharing a common function in different organisms. This initial screen encountered some α-proteobacterial sequence elements that had been previously described, including an *ilvB *leader peptide [20] and long repeat elements [21,22]. Other putative regulatory elements were further evaluated for their potential to form RNA structures by creating a secondary structure model and iteratively searching for additional matches. In the end, we identified five motifs specific to α-proteobacteria that are likely to be structured RNAs (Figure 1).

We experimentally corroborated our secondary structure models for these conserved RNA elements using in-line probing [23]. In this assay, the extent of spontaneous cleavage at each internucleotide linkage in an RNA molecule is determined by separating 5'-radiolabeled degradation products on a polyacrylamide gel. RNA cleavage occurs most rapidly at sites where nucleophilic attack by the 2' oxygen of a ribose approaches an 'in-line' geometry with respect to the phosphorus atom and adjoining 5' oxygen leaving group. Typically, linkages next to base-paired nucleotides in a structured RNA are rigidly held in a conformation that does not permit the formation of an in-line geometry, and therefore these sites cleave slowly. In contrast, internucleotide linkages that are in flexible regions of an RNA molecule occasionally sample an in-line geometry and are cleaved more rapidly. Therefore, regions with relatively low levels of degradation product in an in-line probing gel typically correspond to base-paired or other structured regions of an RNA.

Complete formatted sequence alignments, compilations of downstream genes, consensus structures, and in-line probing data for the five motifs are available (Additional data file 1). Sequence alignments of each RNA motif are also provided in Stockholm format (Additional data files 2, 3, 4, 5, 6) and have been deposited in the Rfam database [24].

### The *serC *element

The short *serC *RNA element (Rfam: RF00517) consists of two conserved base-paired stems. Putative transcription start sites associated with near-consensus upstream promoter elements directly precede all examples of this motif, and the start codon for the *serC *gene is at most 11 nucleotides downstream of the final hairpin. This arrangement suggests that formation of the final hairpin would repress translation by sequestering the ribosome-binding site within the 3' side its base-paired stem and GNRA tetraloop. In-line probing of an RNA corresponding to nucleotides -46 to +11 relative to the *serC *start codon in *A. tumefaciens *(GenBank: NC_003305.1; nucleotides 788249 to 788193) supports this structure.

The *serC *motif is located upstream of an operon encoding serine transaminase (SerC) and phosphoglycerate dehydrogenase (SerA) in many α-proteobacteria. Together, these enzymes convert 3-phosphoglycerate into 3-phosphoserine during the first two steps of serine biosynthesis. SerC can also catalyze a related step in pyridoxal 5'-phosphate (PLP) biosynthesis involving a similar substrate. We have tested whether L-serine, L-threonine, PLP, pyridoxal, pyridoxine, pyridoxamine, or 4-pyridoxic acid are capable of directly binding to the *A. tumefaciens *RNA. None of these compounds have any effect on RNA structure as judged by in-line probing (data not shown). It is possible that an RNA-binding protein could be responsible for sensing a relevant metabolite, binding to the relatively small *serC *element, and derepressing translation. The PyrR protein performs a similar regulatory role for pyrimidine biosynthesis genes in *B. subtilis *[25].

### The *speF *element

The extended *speF *element (Rfam: RF00518) is found upstream of proteins classified into COG0019 in several α-proteobacteria. Primary sequence conservation begins at the 5' end near a putative transcription start site and continues into a base-paired stem that is topped with a large insertion that can form a four-stem junction in some representatives. Following this stem, a stretch of around 80 conserved nucleotides appears to fold into a long bulged stem-loop. This model is tentatively supported by covariation at a few positions in the alignment, except for the outermost putative pairing elements where the sequence is absolutely conserved. The model is also supported by in-line probing patterns for the RNA corresponding to nucleotides -400 to +3 relative to the *speF *translation start site in *A. tumefaciens *(GenBank: NC_003305.1; nucleotides 205774 to 205372). There appear to be further conserved blocks of sequence within the more than 150 nucleotides remaining before the *speF *start codon, but we were unable to assign secondary structures there with much confidence.

Although COG0019 encodes diaminopimelate decarboxylases (*lysA*) in other groups of bacteria, a phylogenetic tree of protein sequences indicates that the genes downstream of this motif are orthologs of *B. subtilis speF*, an ornithine decarboxylase enzyme that catalyzes one of the first steps in polyamine biosynthesis. We have tested whether metabolites related to this pathway bind directly to the *A. tumefaciens *intergenic region and cause structural changes detectable by in-line probing. There is no measurable binding of L-ornithine, L-lysine, *meso*-diaminopimelate, putrescine, cadaverine, or spermidine to the *speF *RNA construct used in this study (data not shown).

### The *suhB *element

The *suhB *element (Rfam: RF00519) was originally recognized upstream of one of nine *A. tumefaciens *ORFs, encoding proteins with similarity to archeal fructose-1,6-bisphosphatases (COG0483). After more matches were found, it became clear that this motif was most likely not a *cis*-acting regulatory element for the *suhB *gene but was more likely to be a small noncoding RNA that is transcribed from the opposite strand relative to the *suhB *gene. In this orientation, each representative carries a putative promoter and intrinsic terminator flanking the conserved sequence domain. Further searches for this motif revealed that multiple copies are present in many α-proteobacterial genomes (for example, five in *Bradyrhizobium japonicum *and four in *Caulobacter crescentus*) and that it is not associated with specific neighboring genes. The only evolutionarily conserved secondary structure in the *suhB *noncoding RNA, aside from the terminator stem, appears to be a short helix near its 5' end. In-line probing of an RNA corresponding to a portion of one *A. tumefaciens *intergenic region containing this motif (GenBank:NC_003305.1; nucleotides 979721 to 979594) also indicates that its characteristically conserved sequences reside in unstructured regions, suggesting that this family could be involved in some form of antisense gene regulation or other noncoding RNA function [26].

### The *ybhL *element

The *ybhL *RNA motif (Rfam: RF00520) appears to be restricted to bacteria from the Rhizobiales. In-line probing data from an RNA corresponding to nucleotides -139 to +21 relative to the translation start site of the *ybhL *gene in *A. tumefaciens *(GenBank: NC_003304.1; nucleotides 2665399 to 2665558) indicate that this element folds into a doubly-bulged hairpin of around 60 nucleotides. Sequence covariation substantiates the formation of the outermost and innermost paired stems. A putative transcription start site is located close to the beginning of the hairpin within a region that appears highly conserved with our limited number of sequence examples. This RNA motif always occurs upstream of genes related to the *Escherichia coli ybhL *gene (COG0670), a putative integral membrane protein. Because the function of *ybhL *is not known, we were unable to formulate any hypotheses for the role of this RNA element.

### The *metA *element

**Figure 2:**
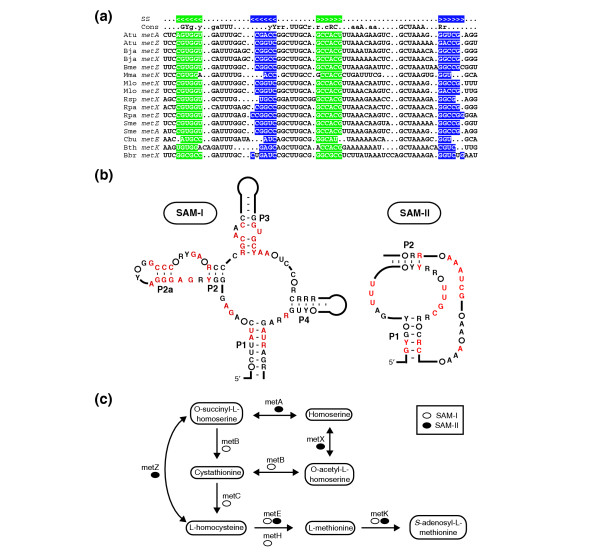
The *metA *RNA element. **(a) **Sequence alignment of representative *metA *RNAs. Shaded nucleotides represent conserved base pairing regions. Lowercase and uppercase letters in the consensus line indicate 80% and 95% sequence conservation, respectively. A complete alignment is available in Additional data file 1. Organism abbreviations: Atu, *Agrobacterium tumefaciens*; Bja, *Bradyrhizobium japonicum*; Bme, *Brucella melitensis*; Mma, *Magnetospirillum magnetotacticum*; Mlo, *Mesorhizobium loti*; Rsp, *Rhodobacter sphaeroides*; Rpa, *Rhodopseudomonas palustris*; Sme, *Sinorhizobium meliloti*; Cbu, *Coxiella burnetii*; Bth, *Bacteroides thetaiotaomicron*; Bbr, *Bordetella bronchiseptica*. **(b) **Consensus sequence and structure of the SAM-I riboswitch aptamer found in Gram-positive bacteria. The consensus is updated from [17] and depicted using the same conventions as Figure 1a. The SAM-II aptamer structure is shown for comparison. **(c) **Comparison of genes in the methionine and SAM biosynthetic pathways found downstream of SAM-I and SAM-II riboswitches.

The *metA *RNA element (Rfam: RF00521) is found in a variety of α-proteobacteria, and there are even a few occurrences in other proteobacterial lineages and the Bacteroides group. This RNA was originally identified upstream of the *metA *gene in *A. tumefaciens*, but was subsequently found preceding other genes related to methionine and *S*-adenosylmethionine (SAM) biosynthesis. The RNA motif is compact with a single stem (P1) and pseudoknot (P2) that are both exceptionally well supported by covariation among more than 70 representatives (Figure 2a). Usually a possible transcription start site with near-consensus -35 and -10 promoter elements is located a few nucleotides before the first nucleotide of P1.

Many representatives also contain putative intrinsic terminators between P2 and the downstream ORF. This transcription terminator arrangement is characteristic of many known riboswitches, and suggests that the *metA *RNA is a regulatory element that functions as a genetic OFF switch [14]. In comparison, Gram-positive bacteria make extensive use of SAM-sensing riboswitches (Figure 2b) to repress a similar collection of methionine biosynthesis genes when SAM becomes abundant in the cell (Figure 2c), often with expression platforms that use transcription termination [15-18]. With consideration of these factors, we tested whether the simpler *metA *motif also functions as a natural aptamer for SAM.

### The *metA *element binds SAM

**Figure 3:**
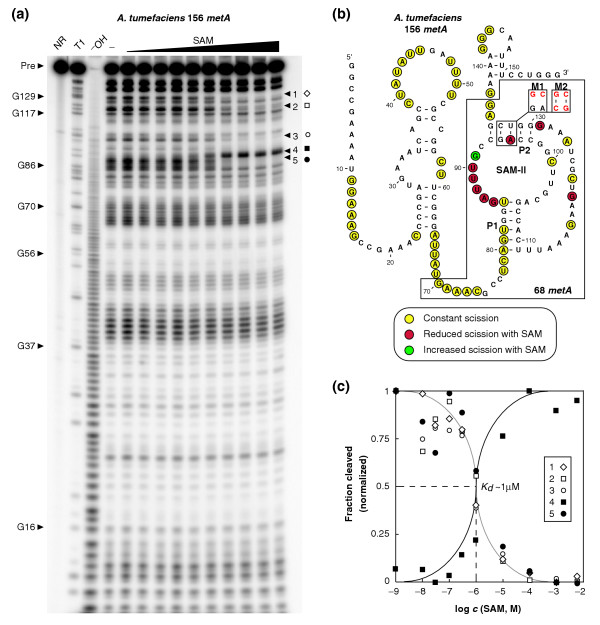
The *metA *element binds SAM. **(a) **In-line probing of 156 *metA *RNA from *A. tumefaciens*. ^32^P-labeled RNA (NR, no reaction) and products resulting from partial digestion with nuclease T1 (T1), partial digestion with alkali (^-^OH), and spontaneous cleavage during a 40 h incubation in the presence of varying of SAM concentrations (1 μM to 6 mM) were separated by polyacrylamide gel electrophoresis. Product bands corresponding to certain G residues (generated by T1 digestion) and full length 156 *metA *RNA (Pre) are labeled. **(b) **Sequence and secondary structure model for *A. tumefaciens metA *RNA. Sites of structural modulation for the 156 *metA *derived from in-line probing are circled with red, green and yellow representing reduced, increased, and constant scission in the presence of SAM, respectively. **(c) **Dependence of spontaneous cleavage in various regions of 156 *metA *on the concentration of SAM. Band intensities for the five regions (labeled 1-5) on the in-line probing gel in (a) were quantitated and normalized to the maximum modulation observed. Data from each of these sites corresponds to an apparent K_d _of around 1 μM (producing half maximal modulation of cleavage) when plotted against the logarithm of the SAM concentration. Theoretical curves for single ligand binding at sites where cleavage increases (black) and decreases (gray) with a K_d _of 1 μM are shown for comparison.

RNA constructs corresponding to nucleotides -230 to -75 relative to the translation start site of the *A. tumefaciens metA *gene (GenBank: NC_003304.1; nucleotides 2703291 to 2703446) were prepared by *in vitro *transcription. The resulting 156-nucleotide RNA (termed 156 *metA*) contains the majority of the intergenic region but excludes the proposed terminator stem. In-line probing assays revealed that the 156 *metA *structure is greatly modulated in response to SAM concentrations ranging from 1 nM to 6 mM (Figure 3a). Mapping spontaneous cleavage patterns onto the secondary structure model for 156 *metA *(Figure 3b) reveals that all SAM-induced changes occur within the conserved *metA *sequence element. There are incidents of both increased and decreased rates of spontaneous RNA cleavage, indicating that SAM does not facilitate general RNA degradation. Rather, SAM associates with 156 *metA *to induce a precise structure that stabilizes certain RNA regions and destabilizes others, as has been seen for all riboswitches characterized previously. An apparent K_d _value of around 1 μM (Figure 3c) for the RNA-SAM complex was determined by plotting the normalized fraction of RNA cleaved in several regions against the logarithm of the SAM concentration.

These results suggested that only the conserved core of this RNA is necessary for SAM recognition. Indeed, a smaller 68-nucleotide *metA *RNA (68 *metA*) encompassing only nucleotides -161 to -94 (GenBank: NC_003304.1; nucleotides 2703360 to 2703426) binds with an affinity of around 10 μM and displays a similar change in its spontaneous cleavage pattern (data not shown). Using 68 *metA*, we examined the importance of the formation of the pseudoknot stem (P2) for SAM binding by making two variants (Figure 3b). One variant carries disruptive mutations (M1: U132→C, C133→G) and the other carries these mutations and the corresponding compensatory mutations (M2: M1, G94→C, A95→G). These RNAs were subjected to in-line probing in the presence of 1 mM SAM (data not shown). Under these conditions, the spontaneous cleavage pattern of M1 did not change in response to SAM. In contrast, M2 exhibited wild-type levels of structural modulation. These results are consistent with covariation in the *metA *sequence alignment that suggests P2 stem formation is required for SAM binding.

We obtained further proof of direct binding between SAM and the *A. tumefaciens metA *RNA by equilibrium dialysis. Adding 10 μM of 156 *metA *to one side of an equilibrium dialysis chamber containing 100 nM *S*-adenosyl-L-methionine-(methyl-^3^H) ([^3^H]SAM), shifted the distribution of [^3^H]SAM to favor the RNA side of the membrane by 2.6-fold. A greater shift was not observed because our [^3^H]SAM sample contained an appreciable amount of radiolabeled breakdown products (see Materials and methods). If 125 μM of unlabeled SAM or the related compound S-adenosyl-L-homocysteine (SAH) are subsequently added to similarly prepared setups, only SAM is able to compete with the [^3^H]SAM and shift the ratio of tritium back to 1. This result demonstrates that 156 *metA *strongly discriminates against the demethylated form of SAM.

The genomic distribution of the *metA *element and its function as a receptor for SAM are consistent with its proposed function as a SAM riboswitch. SAM-II riboswitches found in α-proteobacteria have a consensus sequence and secondary structure that are distinct from SAM-I riboswitches found in the Gram-positive bacteria. A SAM-I riboswitch (the 124 *yitJ *aptamer from *B. subtilis*) has been shown to have a K_d _for SAM of ~4 nM [17]. In contrast, the minimized aptamer from the *A. tumefaciens *SAM-II riboswitch upstream of *metA *has a much poorer affinity for SAM (68 *metA*, K_d _around 10 μM). It has been shown that *in vitro *selected RNA aptamers that have greater information content generally exhibit greater ligand affinity [27]. The SAM-I and SAM-II aptamers follow this general trend, as low-affinity SAM-II aptamers carry two paired elements and only 24 nucleotides that are >80% conserved (Figure 2b). In comparison, SAM-I aptamers incorporate at least four paired stems and 54 conserved nucleotides.

The poorer affinity of the SAM-II aptamer does not necessarily mean that it would exhibit inferior *in vivo *genetic control as a riboswitch. The physiological environments for these riboswitches may be quite different since they operate in divergent groups of bacteria. Furthermore, the kinetics of transcription and ligand binding appear to be more important than equilibrium binding constants for determining whether a flavin mononucleotide (FMN) riboswitch triggers transcription termination [28]. The K_d _for the truncated SAM-II aptamer examined in this study is roughly equal to the SAM concentrations needed to trigger transcription termination by SAM-I riboswitches *in vitro *[15,17]. Furthermore, the affinity of the SAM-II RNA is probably more than sufficient to sense SAM at biologically relevant concentrations. Endogenous SAM levels have been estimated to range from roughly 30 μM to 200 μM in *E. coli *cells grown in rich media [29]. Nevertheless, the ability of the SAM-II motif to function as an efficient riboswitch might be compromised if it were less capable of discriminating against metabolites with structures similar to SAM than the SAM-I aptamer. Therefore, we investigated the molecular specificity of the SAM-II riboswitch in more detail.

### Molecular recognition characteristics of the SAM-II aptamer

**Figure 4:**
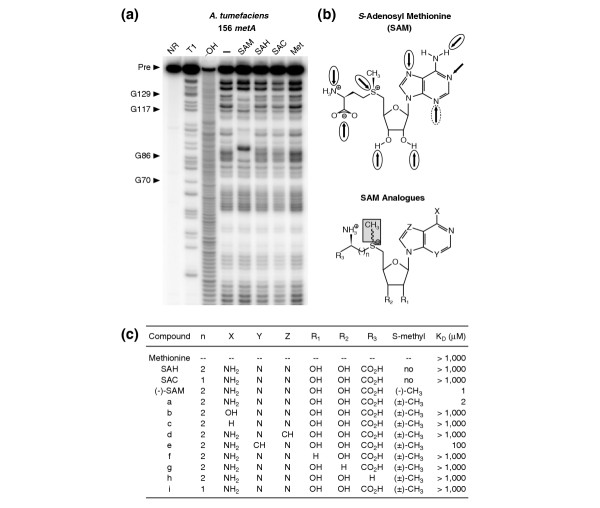
Molecular recognition characteristics of SAM-II aptamers. **(a) **In-line probing of *A. tumefaciens *156 *metA *RNA in the presence of 1 mM SAM, SAH, SAC, and Met. See legend to Figure 3a for an explanation of the labels. **(b) **Chemical structures of SAM and a generalized SAM analogue. Arrows represent possible hydrogen bonds and electrostatic interactions that could serve as points of recognition by the aptamer. Circled interactions were determined to have strong (solid) or weak (dashed) contributions to binding affinity in singly substituted chemical analogues. Recognition of the N1 position of SAM was not tested. **(c) **Apparent K_d _values of SAM analogues for binding to 156 *metA*. Columns (n, X, Y, Z, R_1_, R_2_, R_3_) correspond to groups on the core structure in (b). The *S*-methyl group (gray box) is not present for SAH and SAC.

We performed in-line probing assays with 156 *metA *in the presence of various SAM analogues to measure the discrimination of the SAM-II aptamer against related metabolites (Figure 4). No RNA structure modulation was seen in the presence of 1 mM SAH, *S*-adenosyl-L-cysteine (SAC), or methionine (Figure 4a). A more detailed molecular recognition study (Figure 4b,c) was conducted using a variety of chemically synthesized SAM derivatives (see Materials and methods) containing systematic single substitutions of functional groups that could potentially be recognized by the SAM-II aptamer (compounds *a*-*f*). It is important to note that the biologically active form of SAM used in our initial tests has the (-) sulfonium configuration [30], while the chemically synthesized compounds are racemic (±). Only two of these compounds modulated the riboswitch structure at a concentration of 1 mM. Full titrations indicated that racemic SAM (compound *a*) had a roughly twofold higher K_d _than (-) SAM, and the 3-deaza SAM analogue (compound *e*) bound with a 50-fold higher K_d_.

These analogue binding studies indicate that the SAM-II aptamer creates a binding compartment that recognizes functional groups on the entire surface of SAM. SAM-II discriminates more than 1,000-fold against binding SAM analogues lacking the ribose 2'- or 3'-hydroxyl groups and SAM analogues with single substitutions of the adenine 3-aza, 6-amino, or 7-aza groups. A majority of this affinity loss probably comes from disrupting hydrogen bonds or electrostatic interactions between the aptamer and metabolite, although secondary consequences of the chemical changes, such as altering the preferred ribose sugar pucker or purine ring electronic characteristics, may also contribute to the loss in affinity. Removal of either the carboxyl or amino group from the methionyl moiety is similarly detrimental and might disrupt hydrogen bonds or electrostatic interactions that the aptamer might form with the amino acid zwitterion. Not surpisingly, the aptamer also readily discriminates against the removal of the *S*-methyl group that is critical for the function of SAM as a coenzyme, probably due to the accompanying loss of positive charge on the sulfonium center. Finally, shortening the methionine side chain by one methylene group prevents SAM binding, most likely because it creates a distance constraint that prevents the simultaneous recognition of the methionyl and adenosyl moieties.

We have not investigated whether the 1-aza group of adenine is required for binding, but it is possible that the Watson-Crick face of the adenine base is recognized by a canonical base pair to an aptamer uridine, like that found in the adenine riboswitch [10,31,32]. There are six uracil residues that are absolutely conserved in putatively single-stranded regions of the SAM-II riboswitch and therefore candidates for this interaction (Figure 2b). The molecular recognition determinants for ligand binding by the SAM-II aptamer are depicted in Figure 4b.

The SAM-I riboswitch binds SAH and SAC around 100- and around 10,000-fold poorer than SAM, respectively [17]. The SAM-II aptamer discriminates greater than 1,000-fold against both these compounds, and therefore SAM-II appears to be at least as sensitive to the presence of the *S*-methyl group as SAM-I. Further binding studies with a panel of SAM analogues modified at the sulfonium center indicate that SAM-I tolerates these changes much better than SAM-II (Lim J, Winkler WC, Nakamura S, Scott V, Breaker RR, unpublished data). We are unable to quantitate discriminations of greater than 1,000-fold against analogues for SAM-II due to its poorer overall K_d_. However, our findings indicate that the smaller size of the SAM-II aptamer does not prevent it from attaining the same exquisite discrimination required for efficient genetic control that is exhibited by SAM-I riboswitches.

## Conclusion

Although multiple RNA solutions to small-molecule binding challenges are often found by *in vitro *selection (for example, ATP aptamers; [33-35]), it is now apparent that nature also exploits the structural diversity of RNA to employ multiple, unique mRNA motifs to sense a single metabolite. The SAM-II aptamer found primarily in α-proteobacteria has a much smaller conserved structure than the aptamer of the SAM-I riboswitch from Gram-positive bacteria. Despite having an overall lower affinity for SAM, the SAM-II aptamer appears to be adapted for precise genetic control and discriminates against closely related compounds at least as well as the SAM-I aptamer.

We see two main evolutionary scenarios that could explain the modern phylogenetic distribution of the SAM-I and SAM-II RNAs. SAM, a nucleotide-containing coenzyme, is thought to be a relic of an ancient 'RNA World' when all life processes were controlled primarily by RNA [36-40]. It is possible that RNA World organisms utilized multiple different SAM aptamers for regulatory purposes or as modules incorporated into extinct ribozymes that utilized SAM as a cofactor. According to this hypothesis, the current distribution of each riboswitch might reflect the selective retention of individual classes of SAM aptamers in the progenitors of different bacterial lineages. A second possibility is that the SAM riboswitches emerged more recently and that each aptamer developed independently sometime after the main bacterial lineages diverged billions of years ago [41]. Of course, a combination of ancient and more recent evolutionary events also could account for the distribution of these and other riboswitch classes.

SAM-II is the only known riboswitch that has not been found in the genome of the Gram-positive bacterium *B. subtilis*. We have also identified four other RNA motifs in *A. tumefaciens *that appear to be restricted to other α-proteobacterial genomes. Three of these are candidates for structured mRNA elements, and they join a growing list of putative 'orphan' RNA regulatory elements [14] that might respond to unknown cellular effectors in bacteria. Regardless of the true evolutionary provenance of riboswitches, it is likely that nature employs an even wider diversity of metabolite sensing mRNAs in modern organisms.

## Materials and methods

### Bioinformatics

An updated version of the BLISS database [14,19] containing the results of an all-versus-all BLAST comparison of IGRs from 116 microbial genomes was used to manually examine several α-proteobacterial genomes for conserved RNA elements. The BLISS website displays alignments of homology between bacterial IGRs along with compilations of sequence statistics, species distributions, and neighboring gene function assignments from the COG database [42] in a collaborative annotation environment. The updated version of BLISS is available on the web [19]. Further matches to the five motifs were found by iterative BLAST and filtered covariance model searches [43] of unfinished bacterial genomes and environmental sequences [44]. Phylogenetic trees were constructed with CLUSTALW [45] to clarify the specific functions of some genes assigned to ambiguous COGs.

### In-line probing assays

RNA preparation, radiolabeling, and in-line probing assays were performed essentially as previously described [23]. DNA templates for *in vitro *transcription with T7 RNA polymerase promoters were prepared by whole-cell PCR from *A. tumefaciens *strain GV2260, except for 68 *metA *RNA mutants M1 and M2 where overlapping synthetic oligonucleotides were extended with reverse transcriptase. For each in-line probing reaction, around 1 nM 5' ^32^P-RNA was incubated for 40-48 h in a mixture of 50 mM Tris-HCl (pH 8.3 at 25°C), 20 mM MgCl_2_, 100 mM KCl, and various compounds as indicated. All compounds used for in-line probing were purchased from Sigma. SAM analogues were prepared as diastereomeric mixtures by the reaction of *S*-adenosylhomocysteine derivatives [46,47] and excess methyl iodide [48].

### Equilibrium dialysis

Assays were performed by adding 100 nM *S*-adenosyl-L-methionine-(methyl-^3^H) to side 'a' and 10 μM *metA *RNA to side 'b' of a DispoEquilibrium Biodialyser with a 5 kDa MWCO (The Nest Group, Inc., Southboro, MA, USA) in 40 mM MgCl_2_, 200 mM KCl, 200 mM Tris-HCl (pH 8.5 at 23°C). The sample remaining on side 'a' of the chamber after 10 h of incubation at 23°C was replaced with fresh buffer to increase the final binding signal by preferentially removing non-interacting, radiolabeled metabolite breakdown products [5]. After a second 10 h incubation, the counts in each chamber were recorded. Unlabeled SAM or SAH was added to a concentration of 125 μM in side 'a' and the counts were measured again after a final 10 h incubation.

## Additional data files

The following additional data are available with the online version of this article: A PDF file illustrating the formatted sequence alignments, compilations of downstream genes, consensus structures, and in-line probing data for all five RNA elements (Additional data file 1) and sequence alignments for each of the five RNA motifs in Stockholm format (Additional data files 2, 3, 4, 5 and 6).

## Supplementary Material

Additional data file 1 A PDF file illustrating the formatted sequence alignments, compilations of downstream genes, consensus structures, and in-line probing data for all five RNA elements Click here for file

Additional data file 2 *serC *RNA element sequence alignment in Stockholm format Click here for file

Additional data file 3 *speF *RNA element sequence alignment in Stockholm format Click here for file

Additional data file 4 *suhB *RNA element sequence alignment in Stockholm format Click here for file

Additional data file 5 *ybhL *RNA element sequence alignment in Stockholm format Click here for file

Additional data file 6 *metA *RNA element sequence alignment in Stockholm format Click here for file
