# Supplementary material for: Evidence for a second class of S-adenosylmethionine riboswitches and other regulatory RNA motifs in alpha-proteobacteria
Source: Genome Biol. 2005 Aug 1;6(8):R70. doi: 10.1186/gb-2005-6-8-r70 (PMC1273637; doi:10.1186/gb-2005-6-8-r70)
Supplement: Additional data file 1 — A PDF file illustrating the formatted sequence alignments, compilations of downstream genes, consensus structures, and in-line probing data for all five RNA elements [file gb-2005-6-8-r70-S1.pdf]

Evidence for a second class of *S*-adenosylmethionine riboswitches and other regulatory RNA motifs in alpha-proteobacteria

Keith A. Corbino[*], Jeffrey E. Barrick[†], Jinsoo Lim[*], Rüdiger Welz[*‡], Brian J. Tucker[†], Izabela Puskarz[*], Maumita Mandal[*§], Noam D. Rudnick[*], and Ronald R. Breaker[*]

Addresses: [*]Department of Molecular, Cellular and Developmental Biology, [†]Department of Molecular Biophysics and Biochemistry, and [‡]Department of Chemistry, Yale University, P. O. Box 208103, New Haven, Connecticut 06520-8103, USA.

Current address: [§]Department of Physics, University of California, Berkeley, CA 94720-7200

Correspondence: Ronald R. Breaker. E-mail: ronald.breaker@yale.edu. Phone: 203 432-9389. Fax: 203 432-6604.

Additional Data File 1: Figures S1-S5

(a)

Abb	Organism	Accession/Start-End	Genes
**Alpha Proteobacteria**			
Atu	*Agrobacterium tumefaciens* C58 chr linear	NC_003305.1/788250-788208	*serC* *serA*
Bja	*Bradyrhizobium japonicum* USDA 110	NC_004463.1/8141404-8141361	*serC* *serA*
Bme	*Brucella melitensis* 16M chr I	NC_003317.1/358102-358145	*serC* *serA*
Brs	*Brucella suis* 1330 chr I	NC_004310.1/1631166-1631123	*serC* *serA*
Ccr	*Caulobacter crescentus* CB15	NC_002696.2/3479617-3479574	*serC* *serA*
Mlo	*Mesorhizobium loti* MAFF303099	NC_002678.1/3114177-3114135	*serC* *serA*
Mes	*Mesorhizobium* BNC1	NZ_AAED01000001.1/117537-117580	*serC* *serA*
Mma	*Magnetospirillum magnetotacticum* MS-1	NZ_AAAP01003860.1/22623-22667	*serC* *serA*
Nar	*Novosphingobium aromaticivorans* DSM 12444	NZ_AAAV02000003.1/53466-53509	*serC* *serA*
Rsp	*Rhodobacter sphaeroides* 2.4.1	NZ_AAAE01000117.1/33042-33001	*serC* *serA*
Rpa	*Rhodopseudomonas palustris* CGA009	NC_005296.1/4861073-4861031	*serC* *serA*
Sil	*Silicibacter* TM1040	NZ_AAFG01000007.1/111863-111905	*serC* *serA*
Sme	*Sinorhizobium meliloti* 1021	NC_003047.1/2938127-2938085	*serC* *serA*
**Environmental**			
Env1	Environmental sequence IBEA_CTG_2124392	AACY01024091.1/158-199	*serC*
Env2	Environmental sequence IBEA_CTG_2159813	AACY01068735.1/1559-1517	*serC* *serA*
Env3	Environmental sequence IBEA_CTG_2076514	AACY01075427.1/1481-1439	*serC*
Env4	Environmental sequence IBEA_CTG_2157737	AACY01079269.1/306-348	*serC* *serA*
Env5	Environmental sequence IBEA_CTG_2082756	AACY01092568.1/320-362	*serC* *serA*
Env6	Environmental sequence IBEA_CTG_UBAK909TF	AACY01564294.1/483-441	*serC*

Gene	Description
serC	Phosphoserine transaminase
serA	Phosphoglycerate dehydrogenase

(b)

```
                                                *                                        *
SS    .-35...................-10.................<<<<<<.....>>>>>>....<<<<<<<....>>>>>>>.................
Cons  UUG.........y..................r...gYgGG.cAc.ycycCCcAaCGrgr.gCr...yuaUCUG.aAGGRuag...........AUGry.R.....
Atu   uugacgGAGGCCGGCGACAGGA.auagca.ACGGCGGCAGGACACCUCUCCCUAACGAGAGGCGC..CUAUCUGAAAGGGUAG.UCAA.......augACAGAUAUA
Bja   uugacgACUCUUCCCGUUUCCCGuaucag..CCGCGGCGGGACACCUCCCCCCAACGGGAGGCUUA.CUAUCUGGAAGGGUAG.AAU........augACUGUAGCG
Bme   uugaggAAAAGCGCGCUUUCGGCuauccc..GGUAAGUGGGCCACGUCUCCCCAACGAGACGCGUG.CUAUCUGAGAGGAUAG.AAACAAC....augACGACGAUU
Brs   uugaggAAAAGCGCGCUUUCGGCuauccc..GGUAAGUGGGCCACGUCUCCCCAACGAGACGCGUG.CUAUCUGAGAGGAUAG.AAACAAC....augACGACGAUU
Ccr   uugcaaUUCCCCUCCAGACGCGGuuucuc...GCCGGCGGGCCACGCCCCCCCAACGGGGCGCAGU.CCAUCUGUAAGGAUGG.GAGACAUCGCUaugACCACGACC
Mlo   uugacgCCCGAGGCCUGAACGC.auaucg.CCGUCCGUGGGCCACCCCUCCCCAACGAGGGGCCA..CUAUCUGGAAGGAUAG.ACCACG.....augACGACGCAU
Mes   uugcgcUUUGUCUCCGCUUCGGGuaaugc.CCG.CAGCGGGCUACCCCUCCCCACCGAGGGGCGAG.CUAUCUGGAAGGAUAG.GAACAAC....augACCAUGCUC
Mma   uugcgaAUCGCUCCUGGCCCUC.auacac..UCCGCGCGGGCCACGGUCCCCCAACGGGCCGCGU.CCCUUCUGCGAGGGAGGGUUUUUUAU...augAGCGAUGUU
Nar   uugacaUAUCCCGCCGCGCCAC.uagagc..GACCGGCGGGCCACGCGGUCCCAACGCCGCGCGUG.CUCUCUGCAAGGAGAG.ACUAC......augUCUGCUACG
Rsp   uugcgcCAGAGCCUGCCGGGGCGuaaugg.CGA.CAGCGGGACACCUCUCCCCAACGAGAGGCAUC..UAUCUGGAAGGAUA..CC.........augUCUGAUCUG
Rpa   uugacuUUCGAGGCCAAAGGAC.uauccc..CAGCAGCGGGAAACCUCUCCCCACCGAGAGGCAG..CUAUCUGGAAGGAUAG.ACU........augACUGUAGCG
Sil   uugcgcGGAAUCAUCCUCCCGC.auaucg.CUGUCGGUGGGACACCCCUCCCCAACGAGGGGCGC..UUAUCUGGAAGGGUAG.CAUUU......augGCUAUUGCA
Sme   uugacuUCCUCUCCGAGCCGC..aauauuCCCGGCGGCGGGACACCCCUCCCCAACGAGGGGCUU..CUAUCUGAGAGGAUAG.CAU........augACGAAGCCU
Env1  uuguuaAAGUCUUUCGAAAUC..uauaucCGUUUUUGUGGGACACCCCUCCCCAACGAGGGGCAUA..UAUCUGUAAGGGUA..AAAUAA.....augAAUAUAGAA
Env2  uuguuaAAGGCAGGAUUAUCAA.uuauuu.CGAUACAUGGGACACCUCUCCCCAACGAGAGGCAA..UUAUCUGUAAGGGUAA.CAGAC......augGAUACUAAA
Env3  uugauaAAUGUAAAGUUUUGCAGuauuaa...AGAAAUGGGACAGCUCUCCCCAACGAGAGUCAA..CUAUCUGGAAGGGUAG.AGAAC......augACAAAGAAA
Env4  uugacuAAUCUUAUUAUAAGU..uauauc.UGACUCUUGGGACACUCCUCCCCAACGAGGAGCAA..CUAUCUGUAAGGGUAG.AUAA.......augAUAGAAAAA
Env5  uugacuAAUCUUACUUUAAGU..uauauu.CCACUCUUGGGACACUCCUCCCCAACGAGGAGCAA..CUAUCUGUAAGGGUAG.AGUU.......augAUAGAAAAA
Env6  uugacuAAUCUUAUUAUAAGU..uauauc.UCACUCUUGGGACACUCCUCCCCAACGAGGAGCAA..CUAUCUGUAAGGGUAG.AUAA.......augAUAGAAAAA
```

(c)

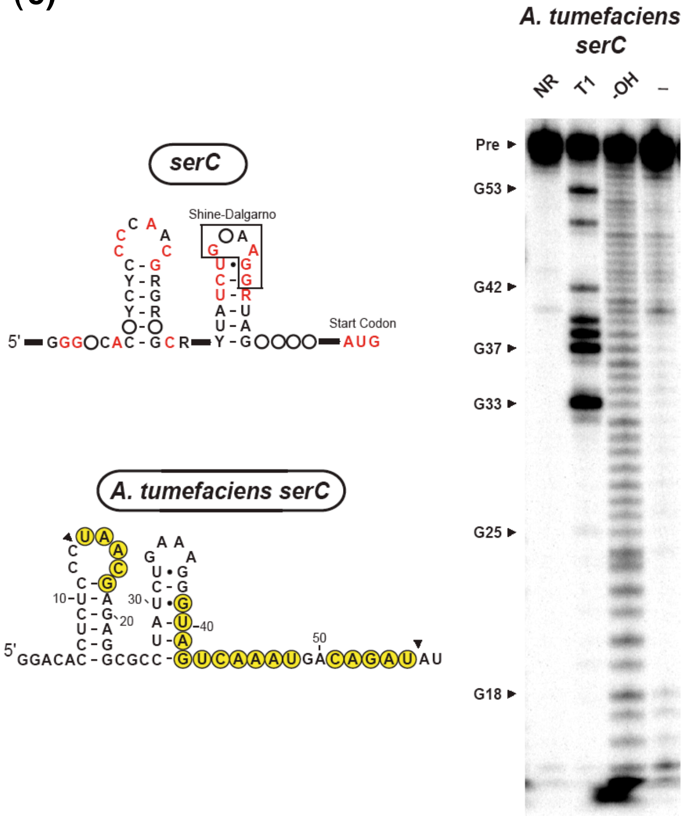

Figure S1

The *serC* Element. **(a)** Species Distribution. Accession numbers refer to GenBank nucleotide records. Functional assignments are provided for the downstream genes that are likely to be regulated if the element is located in the 5′ UTR of an mRNA. **(b)** Sequence Alignment. Labels correspond to abbreviations in (a). Periods represent gaps and dashes indicate the end of a sequence record. The secondary structure line (SS) shows conserved base pairing with angle brackets and shading. The consensus line (Cons) highlights positions with >95% (uppercase) and >80% (lowercase) sequence conservation (R = A, G; Y = C, U). Within each sequence, shaded backgrounds represent base pairing predicted for that individual sequence. Putative elements related to transcription and translation initiation are shown in lowercase letters with shaded backgrounds: orange, promoter -35 and -10 boxes; green, start codons. **(c)** Consensus Structure and In-line Probing. Details for the consensus structure are given in Figure 1. Spontaneous cleavage product in the in-line probing gel are mapped onto nucleotides in the experimental RNA construct probed from *A. tumefaciens* with shaded circles. The boundaries of the region where this mapping was possible are demarked by filled triangles. For the in-line probing gels, the lanes are no reaction (NR), partial RNase T1 digestion (T1), partial alkali digestion ($^{-}$OH), and spontaneous cleavage during a 40 hr incubation (–). The band labeled Pre is the full-length precursor RNA. Some G-specific RNase T1 cleavage products (G18, G20, G22, G23) expected in the T1 lane are missing for *serC*, presumably due to RNA structures that preclude enzyme action.

(a)

Abb	Organism	Accession/Start-End	Genes
**Alpha Proteobacteria**			
Atu	*Agrobacterium tumefaciens* str. C58	NC_003305.1/205774-205416	*speF*
Sme	*Sinorhizobium meliloti* 1021	NC_003047.1/3105445-3105086	*speF*
Bme	*Brucella melitensis* 16M chr II	NC_003318.1/1172829-1172472	*speF*
Brs	*Brucella suis* 1330 chr II	NC_004311.1/97128-97485	*speF*
Mlo	*Mesorhizobium loti* MAFF303099	NC_002678.1/2390868-2390511	*speF*
Rpa	*Rhodopseudomonas palustris* CGA009	NC_005296.1/966567-966102	*speF*
Bja	*Bradyrhizobium japonicum* USDA 110	NC_004463.1/8508267-8508777	*speF*
Bhe	*Bartonella henselae* str. Houston-1	NC_005956.1/1422014-1421658	*speF*
Bqu	*Bartonella quintana*	NC_005955.1/1185319-1184964	*speF*
Mes	*Mesorhizobium* sp. BNC1	NZ_AAED01000003.1/4802-5155	*speF*
Mma	*Magnetospirillum magnetotacticum* MS-1	NZ_AAAP01003568.1/1-285	*speF*

Gene	Description
speF	Ornithine decarboxylase

(b)

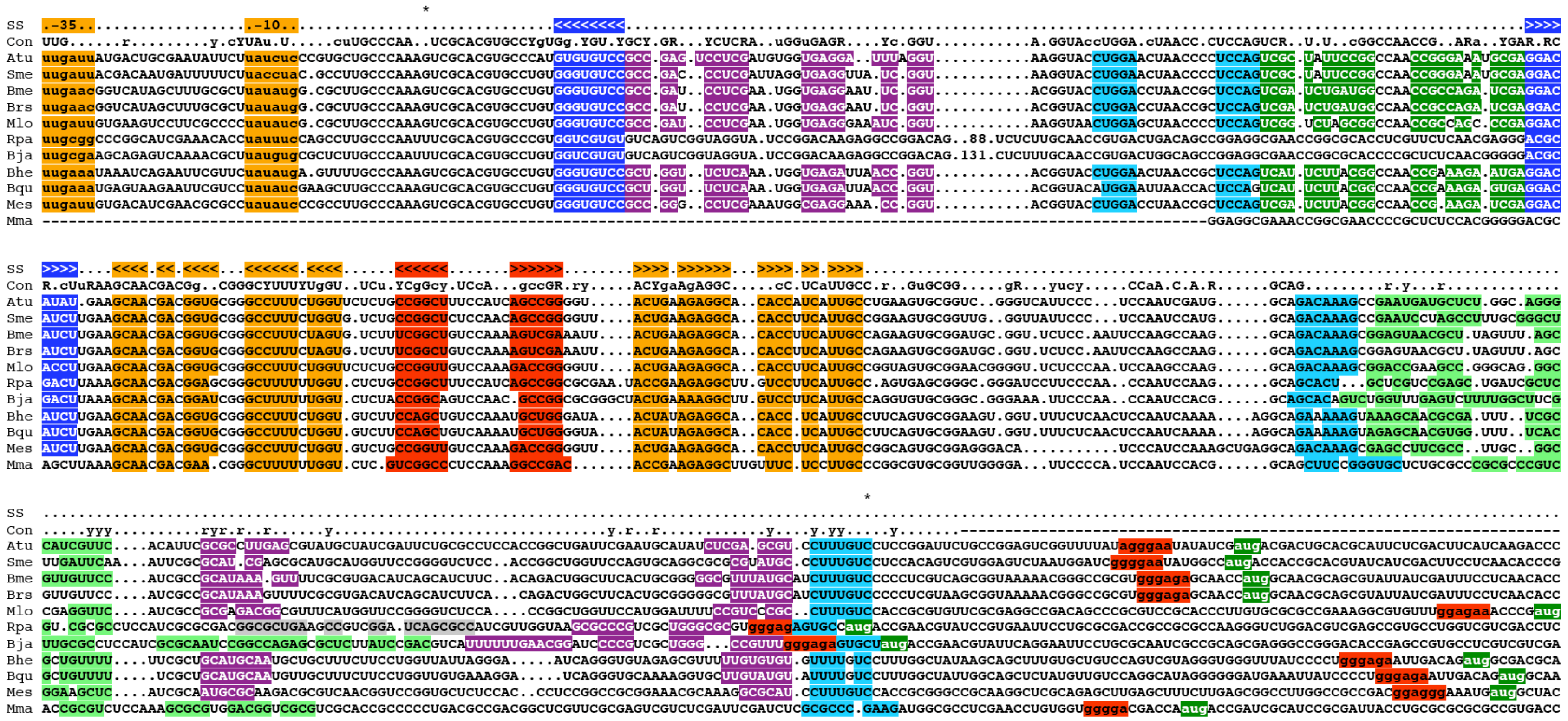

Figure S2 (Page 1/2)

The *speF* Element. **(a)** Species Distribution. Accession numbers refer to GenBank nucleotide records. Functional assignments are provided for the downstream genes that are likely to be regulated if the element is located in the 5′ UTR of an mRNA. **(b)** Sequence Alignment. Labels correspond to abbreviations in (a). Periods represent gaps and dashes indicate the end of a sequence record. The secondary structure line (SS) shows conserved base pairing with angle brackets and shading. The consensus line (Cons) highlights positions with >95% (uppercase) and >80% (lowercase) sequence conservation (R = A, G; Y = C, U). Within each sequence, shaded backgrounds represent base pairing predicted for that individual sequence. Putative elements related to transcription and translation initiation are shown in lowercase letters with shaded backgrounds: orange, promoter -35 and -10 boxes; red, ribosome binding sites; green, start codons.

(c)

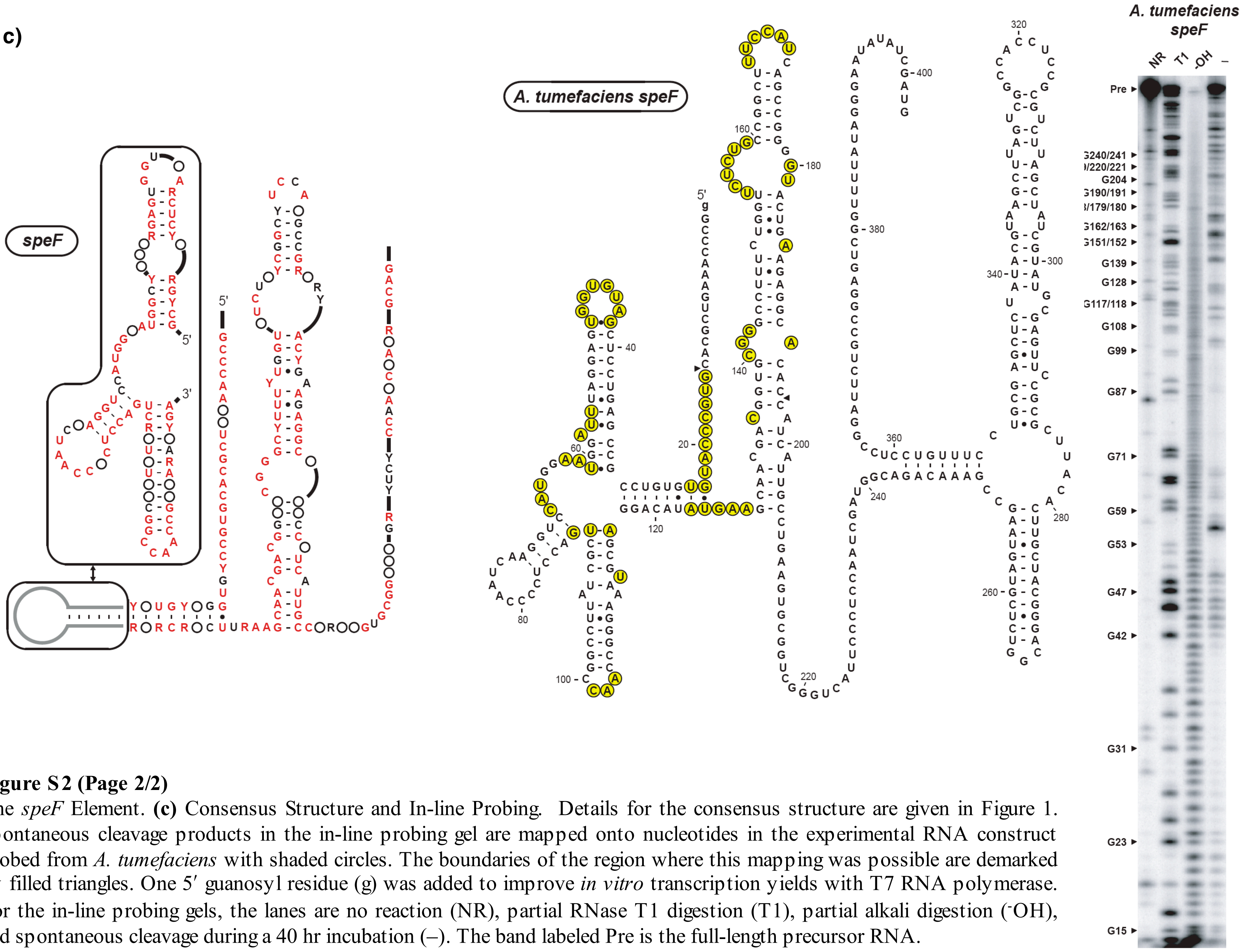

Figure S2 (Page 2/2)

The *speF* Element. **(c)** Consensus Structure and In-line Probing. Details for the consensus structure are given in Figure 1. Spontaneous cleavage products in the in-line probing gel are mapped onto nucleotides in the experimental RNA construct probed from *A. tumefaciens* with shaded circles. The boundaries of the region where this mapping was possible are demarked by filled triangles. One 5′ guanosyl residue (g) was added to improve *in vitro* transcription yields with T7 RNA polymerase. For the in-line probing gels, the lanes are no reaction (NR), partial RNase T1 digestion (T1), partial alkali digestion ($^{-}$OH), and spontaneous cleavage during a 40 hr incubation (–). The band labeled Pre is the full-length precursor RNA.

(a)

Abb	Organism	Accession/Start-End	5′ Gene	RNA	3′ Gene
**Alpha Proteobacteria**					
Atu1	*Agrobacterium tumefaciens* C58 chr circular	NC_003304.1/629693-629653	hypoth. <<	>>	<< COG0128
Atu2	*Agrobacterium tumefaciens* C58 chr linear	NC_003305.1/979638-979604	COG0483 <<	>>	<< COG3741
Bja1	*Bradyrhizobium japonicum* USDA 110	NC_004463.1/1904093-1904129	COG0686 >>	>>	>> COG1145
Bja2	*Bradyrhizobium japonicum* USDA 110	NC_004463.1/2983899-2983858	hypoth. >>	>>	<< COG3125
Bja3	*Bradyrhizobium japonicum* USDA 110	NC_004463.1/3035886-3035840	COG0664 >>	>>	<< COG0477
Bja4	*Bradyrhizobium japonicum* USDA 110	NC_004463.1/7007304-7007265	COG1028 >>	>>	<< COG1028
Bja5	*Bradyrhizobium japonicum* USDA 110	NC_004463.1/8474753-8474708	COG0483 <<	>>	<< COG3741
Ccr1	*Caulobacter crescentus* CB15	NC_002696.2/814142-814181	COG1217 <<	>>	<< COG3741
Ccr2	*Caulobacter crescentus* CB15	NC_002696.2/1221674-1221628	COG0251 >>	>>	<< hypoth.
Ccr3	*Caulobacter crescentus* CB15	NC_002696.2/1676269-1676308	COG0744 <<	>>	<< COG1629
Ccr4	*Caulobacter crescentus* CB15	NC_002696.2/2357586-2357544	COG2755 <<	>>	>> hypoth.
Mma1	*Magnetospirillum magnetotacticum* MS-1	NZ_AAAP01002228.1/65-101		>>	>> hypoth.
Mma2	*Magnetospirillum magnetotacticum* MS-1	NZ_AAAP01003214.1/430-385	hypoth. <<	>>	
Mma3	*Magnetospirillum magnetotacticum* MS-1	NZ_AAAP01003548.1/3056-3094	COG0413 <<	>>	<< COG0526
Mlo1	*Mesorhizobium loti* MAFF303099	NC_002678.1/2445852-2445895	COG0483 <<	>>	<< COG3741
Mes1	*Mesorhizobium* BNC1	NZ_AAED01000003.1/47473-47516	COG0483 <<	>>	<< COG3741
Nar1	*Novosphingobium aromaticivorans* DSM 12444	NZ_AAAV02000001.1/796608-796651	COG3650 >>	>>	>> COG0464
Nar2	*Novosphingobium aromaticivorans* DSM 12444	NZ_AAAV02000008.1/67916-67876	COG1853 >>	>>	>> COG0664
Nar3	*Novosphingobium aromaticivorans* plasmid pNL1	NC_002033.1/78789-78833	COG0629 >>	>>	>> COG4957
Rpa1	*Rhodopseudomonas palustris* CGA009	NC_005296.1/1007361-1007403	COG0483 <<	>>	<< COG3847
Rpa2	*Rhodopseudomonas palustris* CGA009	NC_005296.1/2108276-2108323	COG2333 >>	>>	<< COG1680
Rru1	*Rhodospirillum rubrum*	NZ_AAAG02000001.1/226486-226447	COG0741 <<	>>	<< COG3741
Rru2	*Rhodospirillum rubrum*	NZ_AAAG02000003.1/94-132		>>	>> hypoth.
Sme1	*Sinorhizobium meliloti* 1021	NC_003047.1/3046745-3046780	COG0483 <<	>>	<< COG3741
**Environmental**					
Env1	Environmental sequence IBEA_CTG_2071929	AACY01054089.1/1290-1254	COG0436 <<	>>	>> COG1033
Env2	Environmental sequence IBEA_CTG_2125053	AACY01096949.1/342-306	COG0008 >>	>>	<< COG0477
Env3	Environmental sequence IBEA_CTG_UBAVJ18TR	AACY01598918.1/517-483	COG0436 <<	>>	

COG	Gene	Description
COG0483	*suhB*	Archaeal fructose-1,6-bisphosphatase and related enzymes of inositol monophosphatase family
COG3741	*hutG*	N-formylglutamate amidohydrolase
COG0664	*crp*	cAMP-binding proteins - catabolite gene activator and regulatory subunit of cAMP-dependent protein kinases
COG0436	-	Aspartate/tyrosine/aromatic aminotransferase
COG0477	*proP*	Permeases of the major facilitator superfamily

(b)

```
                                                                                                                        *                                                  *
SS   .-35.....................-10.................<<<<<<<<<<................>>>>>>>>>>...<<<<<<......>>>>>>..........................<<<<<<<<<<<<<<.....>>>>>>>>>>>>>>..........
Cons uug.......................r...........................................................rycy...yyyy...ggry.guu.ucCUCCCy...rA.CUy....g................................yyuuYuuuy..
Atu1 uugccaUCCGCCGCCACCUCGAC..gacgga.34.GGGAAGGCGACGCCCAGGAAGACGAACAGCGCGAGAAGUCUUGCGGCGAUGGUCU...UCAC...GGGU.GAU.CCCUCCCUAAUGG.CUUCA..GUGCUGC.......GGCUCG.......GCAGCGCUUUCCUUUUC
Atu2 uugacaAUUGUGCAGUUACUGCG..uagcgu...........GACCCUGCAGUC........UUU.........GACUGUAAAUACCC..UCCUU...GGGU.GUU.UCCUCCCU..AGA.CUUGA..CCGCGC.........UCAG.........GCGCGGUUUUUUUUGC
Bja1 cuaacaUACAAGGCGGUGGCCGA..gagccu......CGGGCUGCCCUUCUCCCCGAUCGAACAGGCUGCGGCCUGAUCCCA.GAGGCCC...UCAU...GGGC.GUU.UCCUCCCU...GA.CUAA...GGCCACCC.......UUCG.......GGGUGGCUCUUUUUUUCG
Bja2 uugaacUCCGUCGGGAGUUGCUUAGcuuaua.27.UUCCUUGGCAGCAAUCCG...........GUU...........CGGAU..UGCUU..UGCUC...GGGC.GUU.UCCUCCCU..AGA.CUUGG..GCCGCUUGU......CACAA.....ACAAGCGGCUCUUCUUUUU
Bja3 uucacaCCCAGGGCAUUCUCGGCU.ucuccg........GCGCCCGCCAGAUCGUGCUGCGCAACCGCCAGCGCCUGCACAAUCUCGACG..CCUGAU..CGUC...U.UCCUCCCCACCCA.CU.....UGGCCGGCCGAA...CCCGU..UUCGGCCGGCCAUUUCUUUUGC
Bja4 uagacaCACACUCUCGGGUCGUGCCgauauc.......................CAUUUCAAGACCGAGAUGACCGAACCCCUUGCAGAC..CGGCU...GUCU.GUU.UCCUCCCG...AA.CUU....GGGCGCCGUC.....UAGA.....GGCGGCGCCCAUUCUUUCGG
Bja5 uugcacUUUGUGCGGCGCGGCCGC.auauug..........UUGCGGUGCGGUAGCGC......UCU......GCGUUACCGC..UGCCC..UCCUU...GGGC.GUU.UCCUCCCU..AGA.CUU....GGGCCGCUUGCG...UCAUU..CGCGAGCGGCCCUUUUUUCUUU
Ccr1 uugccuAAUUUUGCGCCGCACUC..uaaaaa........CAAUCCUGUGAGGCGGC........GCU........GCCGCUUC..UGCCC..UUCCU...GGGC.GUU.UCCUCCCUAAUGAACUG....GCCCGGCG.......GUUU.......CGCCGGGCUUUUUUUUGG
Ccr2 uugucaAGGCGAGGUCAAGUAGC..uauaug..................................GAACUC...AGCCC...UUUC...GGGC.GUU.UCCUCCCA.AUGA.CU.....CGGCCGCCCUCU..UCUCCU..GGAGGGCGGCCGUUCUUUUGUC
Ccr3 uugaacACCCGAAAAGUGAAC....auagau........CACCAACGUGAGGCU..........GAG..........AGCCUC..UGCCC..UUCCUG..GGGC.GUU.UCCUCCCUACUUA.UUUCA..GCCCGGC.......UUUUC......GCCGGGCUUUUCUUUUG
Ccr4 uugcaaCUGGACCUGUGUUAGCGC.uaucaa.........UCGCGUGUGAGGCGC.........CGA.........GCGCCUC..UGCCC..UUCCU...GGGC.GUU.UCCUCCCU.AUGA.CUUUGUAGCCCGGUC.......UUCAU.....GGCCGGGCUUUCUUUUUG
Mma1 --------------------------------10.GAUCAUACAAGCAUACGAGGUCGCG..AUGGCG...UGCGGCC.CGUA...GCCC...UUCU...GGGC.GUU.U.CUCCCU..AGA.CUUCGG.GCCGUCC.......UUCG.......GGGCGGCUUUUUCUUUC
Mma2 uucccaGGAGCGCCGCCGAUCACC.gaccau........CAUCCGGCACGGUCCU.......UGCUGGC.......AGGUCCG.AUGCCC..AUGGC...GGGC.GUU.UCCUCCCU..UGA.CUUC...GGCCCCGC.GCG...ACGAC..CGCUGCGGGGCCUUUUUUUCGG
Mma3 gugcccAUCACGCGGAACGGC....cgacau.12.GAUCCCGUGAGGUGCGCG........CCGCUGGCC.........CCGCU..CGCCC..UUUUUC..GGGC.GUU.UCCUCCCU..AGA.CUU....CGGUCCGCC......UCCG......GGCCCGCCGCUUUUCCGCC
Mlo1 uugacaUUUGUGCACCGCACAA...uacccu..........CGACCUCGGACAGG........GUUU........CCUGUCC.AUGCCC..UCCUU...GGGC.GUU.UCCUCCCU..AGA.CUUC...GACCGUAUCGU....GCAA....ACGAUGCGGUCUUUUUUUACA
Mes1 uugacaGUUGUGCAAUGCAGCA...uagacu..........UUGAUUCGGACAG.........UGACC.........CUGUCC.AUGCCC..AUCUU...GGGC.GUU.UCCUCCCU..AGA.CUUU...GACCGUGUCGU....GAAA....ACGAUACGGUCUCUUUUUAAA
Nar1 uugaccCAUAAAUUGCAAAAAC...ugcaacGAGGAUUUCUCAUUUCGCACUUGCACAAAAACAGGGUGCGUUGCAUACAGAUUCUGCCU...UUC....AGGC.AUC.CUCUCCCA..AAA.CUUUCC.GACCCGGCGG.....GCAA.....CUGCCGGGUCUUUUUUUUGU
Nar2 uugacaUCCGUCAACGCGCGGCCG.gacgguCGCGCCUAAUCCUGACCUCGACGAAGCGGGAACAAGACCUCACCCCCGGACACCGCC..CCGCUUC...GGC.GAU.UUCUCCCC...AA.CUU....GGGCGGCCG....UGCAGUC.....UGGCCGCCCUAUUUUUUUG
Nar3 uugcagCAUAGGUGCCCGCGACC..cauauc..............................AGGCUUGCCU...UUC....AGGC.AUC.CUCUCCCA..AGA.CUUUCACGGGGCCGGCA.....GCAA.....UGCUGGCCCCAUUUUUUUGU
Rpa1 cugacaUGCGUUUUUUGCAUG....gaaaau.38.UAUUGUUGCGGUGCGGUAGCGC......CUC......GCGCUAUCGC..UGCCC..UCCUU...GGGC.GUU.UCCUCCCU..AGA.CUU....GGGCCGCUUGC....CAUC....GCAAGCGGCCCUUUUUUCUUU
Rpa2 uugcccAAAUAGAACGCACGC....uagaag..6.ACAUACUGAACAUCCGCGAUG......CGGUU......CGUCGCGG..UCCCC..UUCCUU..GGGGCGUU.UCCUCCCU..AGA.CUU....G.GGCCCGGGCGCC.GAAU.GGCGACCGGGCCUCUUUAUUUGUC
Rru1 uugccaAGAGCGGGGGCGCUCCCU.aaauuu...................UGCGAUGCAGCACGAGGCGCAUCUGUGCCAAGAGCUG.CU...UCUU...GGAC.GUU.UCCUCCCU..AAA.CUC....GGGCCGUGG.....UUUUU.....CCACGGCCCCUUUUUCUGU
Rru2 --------------------------------29.GCAUAUUUUGCAGCGCAGCAUGACGGUCCAUGCGUUUUUCGCAGUUAUGCUGUCU..UUCUU...GGAC.GUU.UCCUCCCU..GAA.CUU....GGGCCGUAG......GUAA......CUACGGCCCCUUUUUUUCG
Sme1 uugacaAUUGUGCACCGCAGCAU..uacagu...........UUUUCUGCAGUCA.......CUCG........UGGCUGCAAAUACCC..UCCUU...GGGU.GUU.UCCUCCCU..AGA.CUUGA..CCGCGCC........GCU........GGCGCGGUUCUUUUUGA
Env1 augacaGCAUUGCUACUAUCACA..uaucgg.38.CUAUAUCAGGGCUGUUGAU.........AUUU.........AUCAAC..CCUUCGAUUUUU.UCGAGG.GUU.UCCUCCCU..AAA.CUUAA..GCCACU........CUUAGC........AGUGGCUUUUUUUUUAC
Env2 uugacaGAAUCGUGCAAUAUGC...uauuaa..............................UGCAUUGCAAAAUAA..UUUUU...UUAU.AUU.UCCUCCCU..GAA.CUCA...GCUGCAA.......UUAAU.......UUGCAGCUUUUUUUUUAA
Env3 augccaGCAUUGUGAUUCUUACA..uaucgg.39.CUAUAUCAGGCCUGUUGAU.........CUUU.........AUCAAC..CCCUCGCUUAUUACCGAGG.GUU.UCCUCCCU..AAA.CUUGA..GCCACU........UUAA........AGUGGCUUUUUUUUUGC
```

Figure S3 (Page 1/2)

The *suhB* Element. **(a)** Species Distribution. Accession numbers refer to GenBank nucleotide records. Relative directions and general functional assignments according to the COG database are provided for the adjacent upstream and downstream genes. **(b)** Sequence Alignment. Labels correspond to abbreviations in (a). Periods represent gaps and dashes indicate the end of a sequence record. The secondary structure line (SS) shows conserved base pairing with angle brackets and shading. The consensus line (Cons) highlights positions with >95% (uppercase) and >80% (lowercase) sequence conservation (R = A, G; Y = C, U). Within each sequence, shaded backgrounds represent base pairing predicted for that individual sequence. Putative promoter -35 and -10 boxes are shown as lowercase letters with orange backgrounds.

(c)

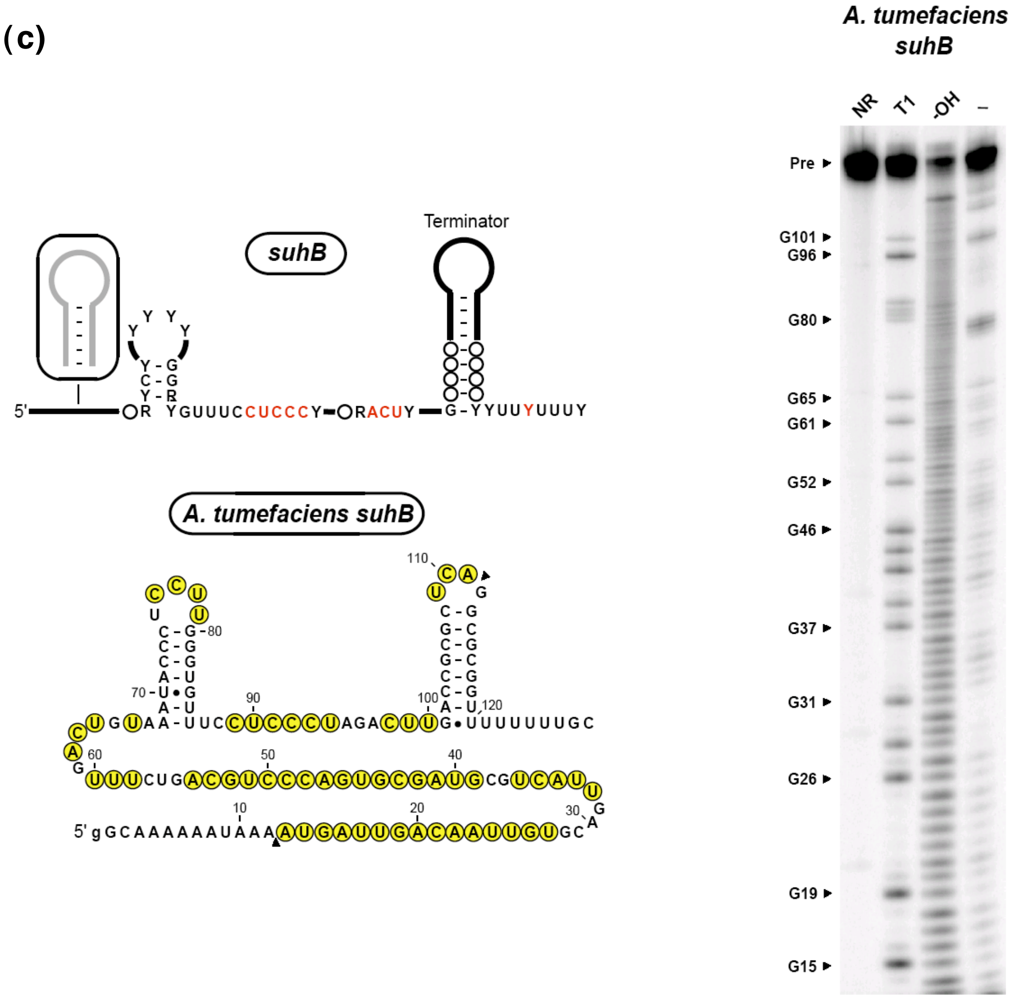

Figure S3 (Page 2/2)
The *suhB* Element. **(c)** Consensus Structure and In-line Probing. Details for the consensus structure are given in Figure 1. Spontaneous cleavage products in the in-line probing gel are mapped onto nucleotides in the experimental RNA construct probed from *A. tumefaciens* (Atu2) with shaded circles. The boundaries of the region where this mapping was possible are demarked by filled triangles. One 5′ guanosyl residue (g) was added to improve *in vitro* transcription yields with T7 RNA polymerase. Note that the initial stem appears to be extended by three closing base pairs compared to the consensus structure in this particular sequence and, as is often seen in intrinsic transcription terminators, two uridines of the final polyU run seem to pair with nucleotides directly 5' of the G-C rich stem. For the in-line probing gels, the lanes are no reaction (NR), partial RNase T1 digestion (T1), partial alkali digestion ($^{-}$OH), and spontaneous cleavage during a 40 hr incubation (–). The band labeled Pre is the full-length precursor RNA.

(a)

Abb	Organism	Accession/Start-End	Genes
Alpha Proteobacteria			
Atu	*Agrobacterium tumefaciens* str. C58 chr circular	NC_003304.1/2665461-2665537	COG0670
Bme	*Brucella melitensis* 16M chr I	NC_003317.1/1909433-1909342	COG0670
Brs	*Brucella suis* 1330 chr I	NC_004310.1/93635-93726	COG0670
Mlo	*Mesorhizobium loti* MAFF303099	NC_002678.1/3454185-3454105	COG0670
Mes	*Mesorhizobium* BNC1	NZ_AAED01000002.1/530436-530361	COG0670
Sme	*Sinorhizobium meliloti* 1021	NC_003047.1/3506027-3506125	COG0670

COG	Gene	Description
COG0670	*ybhL*	Integral membrane protein, interacts with FtsH

(b)

```
                                            *
SS  .-35.....................-10.................<<<<<<<<....<<<....<<<<<<<<<<
Con UUGu.Y.yga.r......a.C.uCAUAuU...y.rcAgGCaUGCUGgagYYCCG.CAGCGGCGccYGG........
Atu uuguauCGGACACGUUCUGCCAUCAuauucgUGAGUAGGCUUGCUGAAGCUCCG.CAGCGGCGCCCGGA......
Bme uuguacGCGAGGGUUGAAAUCCCCAuaaugaAUCACAGGCAUGCUGGGGUCCCGCCAGCGGCGCCUGGCGGCAGCU
Brs uuguacGCGAGGGUUGAAAUCCCCAuaaugaAUCACAGGCAUGCUGGGGUCCCGCCAGCGGCGCCUGGCGGCAGCU
Mlo uuguucUUGACGCGAAUAACCAUCAuauuucGCCACAGGCAUGCUGGAGCCCCGCCAGCGGCGCCUGGGUC.C...
Mes uugcccUUGCACCAAACAAACAUCAuauuca.CACCACGCAUGCUGGACUCCCGCCAGCGGCGAGCGGCG......
Rle uuguuuGCAAAGGGCGAAAGCCUCAuauuau.CCGCAGGCAUGCUGGAGCUCCG.CAGCGGCGCCCGGG.U.....
Sme uugugcAUGACGGCGUUCACCAUCAuauuugUCCACAGGCAUGCUGGAGCCCCG.CAGCGGCGCCUGGGCGCGCAA

                                                                          *
SS  <<<........>>>>>>>>>>>>>....>>>..........>>>>>>>>........................
Con .....................cCCGACA.CCG.A........CGGRRc..rr......aaGAGGA........AUG
Atu .....GGUA...........UCCCGACA.CCGUAGCAAUCA.CGGAGCUCAA......aagaggAAACA....aug
Bme UGU..GCAU...AUGAGC.GCCCCGACA.CCGGA........CGGGACAAGGC.....aagaggAUUGAAGUCaug
Brs UGU..GCAU...AUGAGC.GCCCCGACA.CCGGA........CGGGACAAGGC.....aagaggAUUGAAGUCaug
Mlo .....GUAA.........GAGGCCCGACA.CCGGA........CGGGGCAGAAGCAA..uagaggAUUCCA...aug
Mes ......UUG...........UGCCGACACCCG.A........CGGGAGAGUAGCUAGAaagaggAACAC....aug
Rle .....CGAAA.........ACCCCGACA.CCGUA.UUUCCAUCGGAGCUUGU......aagaggAAACU....aug
Sme AGGAACGACAAGG.UUCGCCGCCCGACA.CCGUAACUUCAACCGGGGCUUAA......augaggAAACA....aug
```

(c)

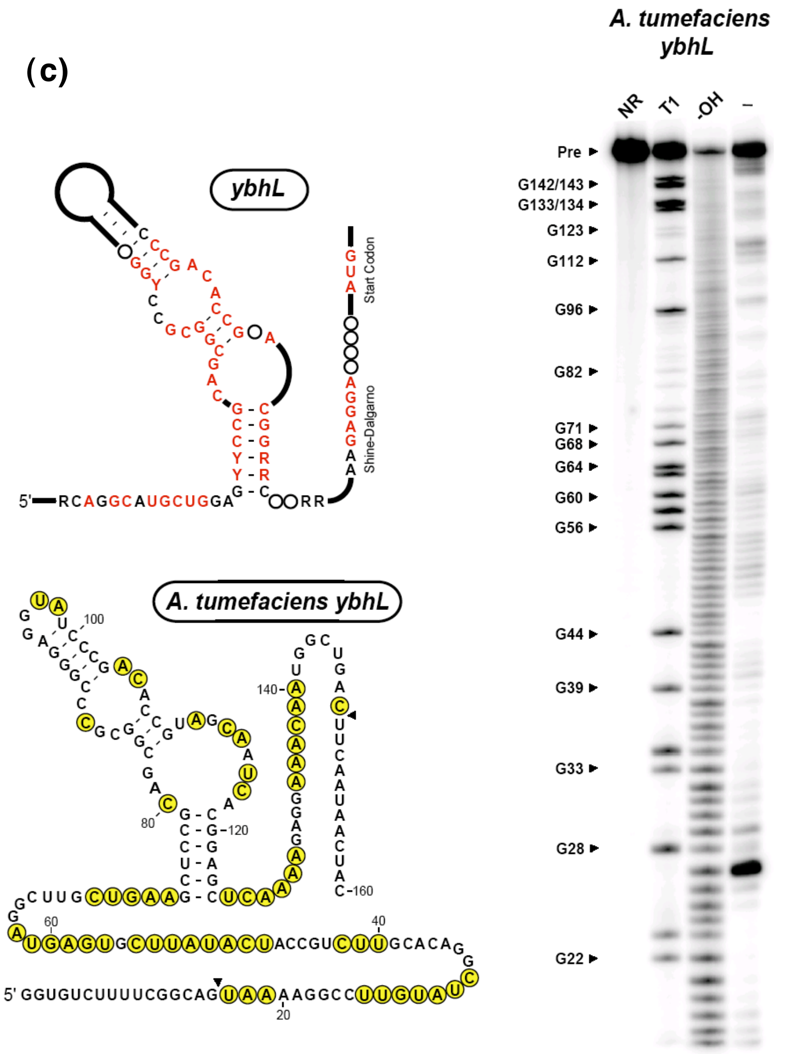

Figure S4

The *ybhL* Element. **(a)** Species Distribution. Accession numbers refer to GenBank nucleotide records. Functional assignments are provided for the downstream genes that are likely to be regulated if the element is located in the 5′ UTR of an mRNA. **(b)** Sequence Alignment. Labels correspond to abbreviations in (a). Periods represent gaps and dashes indicate the end of a sequence record. The secondary structure line (SS) shows conserved base pairing with angle brackets and shading. The consensus line (Cons) highlights positions with >95% (uppercase) and >80% (lowercase) sequence conservation (R = A, G; Y = C, U). Within each sequence, shaded backgrounds represent base pairing predicted for that individual sequence. Putative elements related to transcription and translation initiation are shown in lowercase letters with shaded backgrounds: orange, promoter -35 and -10 boxes; red, ribosome binding sites; green, start codons. **(c)** Consensus Structure and In-line Probing. Details for the consensus structure are given in Figure 1. Spontaneous cleavage products in the in-line probing gel are mapped onto nucleotides in the experimental RNA construct probed from *A. tumefaciens* with shaded circles. The boundaries of the region where this mapping was possible are demarked by filled triangles. For the in-line probing gels, the lanes are no reaction (NR), partial RNase T1 digestion (T1), partial alkali digestion (⁻OH), and spontaneous cleavage during a 40 hr incubation (–). The band labeled Pre is the full-length precursor RNA.

(a)

Abb	Organism	Accession/Position	Genes	
**Alpha Proteobacteria**				
Atu1	*Agrobacterium tumefaciens* C58 chr circular	NC_003304.1/429424-429378	COG0626	
Atu2	*Agrobacterium tumefaciens* C58 chr circular	NC_003304.1/2703363-2703434	COG1897	
Bja1	*Bradyrhizobium japonicum* USDA 110	NC_004463.1/1219858-1219690	COG0626	
Bja2	*Bradyrhizobium japonicum* USDA 110	NC_004463.1/1515006-1515379	COG2021	COG0500
Bja3	Bradyrhizobium japonicum USDA 110	NC_004463.1/7516089-7516237	COG0639	
Bme	*Brucella melitensis* 16M chr I	NC_003317.1/1664142-1664216	COG0626	
Brs	*Brucella suis* 1330 chr I	NC_004310.1/318415-317990	COG0626	
Mma1	*Magnetospirillum magnetotacticum* MS-1	NZ_AAAP01003855.1/12513-12446	COG0192	COG0220
Mma2	*Magnetospirillum magnetotacticum* MS-1	NZ_AAAP01003864.1/43184-43111	COG0626	
Mlo1	*Mesorhizobium loti* MAFF303099	NC_002678.1/2853515-2853846	COG2021	COG0500
Mlo2	*Mesorhizobium loti* MAFF303099	NC_002678.1/4543368-4543296	COG0626	
Mes1	*Mesorhizobium* BNC1	NZ_AAED01000001.1/195260-195332	COG1897	
Mes2	*Mesorhizobium* BNC1	NZ_AAED01000008.1/69545-69618	COG0626	
Rsp	*Rhodobacter sphaeroides* 2.4.1	NZ_AAAE01000150.1/27825-27754	COG0626	
Rpa1	*Rhodopseudomonas palustris* CGA009	NC_005296.1/5009663-5009589	COG2021	COG0500
Rpa2	*Rhodopseudomonas palustris* CGA009	NC_005296.1/5380828-5380777	COG0626	
Rru	*Rhodospirillum rubrum*	NZ_AAAG02000001.1/970168-970115	COG0626	
Sme1	*Sinorhizobium meliloti* 1021	NC_003047.1/580264-580068	COG0626	
Sme2	*Sinorhizobium meliloti* 1021	NC_003047.1/3461576-34618381	COG1897	
**Beta Proteobacteria**				
Bbr	*Bordetella bronchiseptica* RB50	NC_002927.3/4852879-4852804	COG2021	COG0500
Bpa	*Bordetella parapertussis* 12822	NC_002928.3/4417692-4417617	COG2021	COG0500
**Gamma Proteobacteria**				
Cbu	*Coxiella burnetii* RSA 493	NC_002971.2/1955247-1955190	COG0620	COG0685
**Bacteroides**				
Bth	*Bacteroides thetaiotaomicron* VPI-5482	NC_004663.1/4108979-4109039	COG0192	
Pgi	*Porphyromonas gingivalis* W83	NC_002950.2/1994581-1994639	COG0192	

COG	Gene	Description
COG2021	***metX***	Homoserine acetyltransferase
COG0646	***metH_1***	Methionine synthase I (cobalamin-dependent), methyltransferase domain
COG1410	***metH_2***	Methionine synthase I, cobalamin-binding domain
COG0626	***metC***	Cystathionine beta-lyases/cystathionine gamma-synthases
COG1897	***metA***	Homoserine trans-succinylase
COG0500	***bioC***	SAM-dependent methyltransferases
COG2873	-	O-acetylhomoserine sulfhydrylase
COG0192	***metK***	S-adenosylmethionine synthetase [metK]
COG0155	***cysI***	Sulfite reductase, beta subunit (hemoprotein)
COG0220	-	Predicted S-adenosylmethionine-dependent methyltransferase
COG0639	***apaH***	Diadenosine tetraphosphatase and related serine/threonine protein phosphatases
COG0620	***metE***	Methionine synthase II (cobalamin-independent)
COG0685	***metF***	5,10-methylenetetrahydrofolate reductase

Abb	Organism	Accession/Position	Genes	
**Environmental Sequences**				
Env01	IBEA_CTG_2058111	AACY01000458.1/1106-1037	COG2021	
Env02	IBEA_CTG_2083395	AACY01001152.1/1304-1233	COG0646	COG1410
Env03	IBEA_CTG_2069629	AACY01013609.1/1550-1623	COG2873	
Env04	IBEA_CTG_2079783	AACY01015393.1/1522-1452	COG0646	COG1410
Env05	IBEA_CTG_2092782	AACY01015394.1/1298-1227	COG0646	COG1410
Env06	IBEA_CTG_1978138	AACY01019662.1/986-1056		
Env07	IBEA_CTG_2068602	AACY01020820.1/545-476	COG0155	
Env08	IBEA_CTG_2043976	AACY01037785.1/1397-1325	COG2873	
Env09	IBEA_CTG_2153715	AACY01038093.1/573-642	COG2021	
Env10	IBEA_CTG_2147227	AACY01048873.1/713-779	COG2021	
Env11	IBEA_CTG_2155094	AACY01075589.1/2322-2395	COG2873	
Env12	IBEA_CTG_1961583	AACY01076673.1/123-192	COG2021	
Env13	IBEA_CTG_2151957	AACY01076967.1/2586-2515	COG2021	
Env14	IBEA_CTG_2012917	AACY01078968.1/460-390	COG2021	
Env15	IBEA_CTG_2157446	AACY01080761.1/1069-999	COG2873	
Env16	IBEA_CTG_2159957	AACY01095680.1/1999-1930	COG2021	
Env17	IBEA_CTG_2149224	AACY01101458.1/5920-5851	COG2021	
Env18	IBEA_CTG_2096709	AACY01103076.1/1423-1354	COG2021	
Env19	IBEA_CTG_2150356	AACY01108759.1/4012-3939	COG2873	
Env20	IBEA_CTG_2118022	AACY01120549.1/929-862	COG2021	
Env21	IBEA_CTG_1956900	AACY01121489.1/451-524	COG2873	
Env22	IBEA_CTG_2027402	AACY01135886.1/285-216	COG2021	
Env23	IBEA_CTG_2037958	AACY01137881.1/882-811	COG2873	
Env24	IBEA_CTG_2064653	AACY01143447.1/813-880	COG0646	
Env25	IBEA_CTG_SKAQM84TR	AACY01181281.1/445-378	COG2021	
Env26	IBEA_CTG_SKATY55TR	AACY01185668.1/363-291	COG0626	
Env27	IBEA_CTG_SKBKZ58TF	AACY01206430.1/333-401	COG2873	
Env28	IBEA_CTG_SKBT336TR	AACY01216447.1/333-263	COG2021	
Env29	IBEA_CTG_SLAO627TF	AACY01230350.1/529-460	COG2021	
Env30	IBEA_CTG_SLBMZ82TF	AACY01253705.1/36-105	COG2021	
Env31	IBEA_CTG_SSANF95TR	AACY01273405.1/372-439	COG2021	
Env32	IBEA_CTG_SSBBN24TR	AACY01293033.1/913-844	COG2021	
Env33	IBEA_CTG_SSBFE56TR	AACY01303516.1/371-301	COG2021	
Env34	IBEA_CTG_SSBFL24TR	AACY01304150.1/460-391	COG0646	
Env35	IBEA_CTG_SSBH615TF	AACY01309385.1/703-634	COG2021	
Env36	IBEA_CTG_SSBTD84TR	AACY01348042.1/440-510	COG2021	
Env37	IBEA_CTG_SSBUT42TR	AACY01352090.1/431-373	COG2021	
Env38	IBEA_CTG_SSBW488TR	AACY01356025.1/783-713	COG0646	
Env39	IBEA_CTG_SSBWL94TF	AACY01357550.1/484-553	COG2021	
Env40	IBEA_CTG_SXAA622TF	AACY01372079.1/300-369	COG2021	
Env41	IBEA_CTG_SZAFX30TR	AACY01401139.1/470-401	COG2021	
Env42	IBEA_CTG_SZAIM36TR	AACY01409674.1/455-524	COG2021	
Env43	IBEA_CTG_SZAT770TR	AACY01441579.1/352-283	COG2021	
Env44	IBEA_CTG_UAAAS64TR	AACY01453671.1/550-479	COG2021	
Env45	IBEA_CTG_UAALM53TF	AACY01485766.1/38-109	COG0646	
Env46	IBEA_CTG_UAAOF79TR	AACY01494096.1/183-249	COG0626	
Env47	IBEA_CTG_UAARI50TF	AACY01503107.1/98-169	COG0646	
Env48	IBEA_CTG_UBAAV22TR	AACY01536804.1/768-697	COG0646	
Env49	IBEA_CTG_UBAYI05TR	AACY01607341.1/466-537	COG0646	
Env50	IBEA_CTG_UDASE14TF	AACY01690877.1/292-222	COG2021	
Env51	IBEA_CTG_UDAT846TF	AACY01693958.1/438-508	COG0646	
Env52	AMC_Cont262	AADL01000262.1/158-228		

Figure S5 (Page 1/3)

The α-Proteobacterial SAM Riboswitch. **(a)** Species Distribution. Accession numbers refer to GenBank nucleotide records. Functional assignments are provided for the downstream genes that are likely to be regulated if the element is located in the 5' UTR of an mRNA. **(b)** Sequence Alignment. Labels correspond to abbreviations in (a). Periods represent gaps and dashes indicate the end of a sequence record. The secondary structure line (SS) shows conserved base pairing with angle brackets and shading. The consensus line (Cons) highlights positions with >95% (uppercase) and >80% (lowercase) sequence conservation (R = A, G; Y = C, U). Within each sequence, shaded backgrounds represent base pairing predicted for that individual sequence. Putative elements related to transcription and translation initiation are shown in lowercase letters with shaded backgrounds: orange, promoter -35 and -10 boxes; green, start codons.

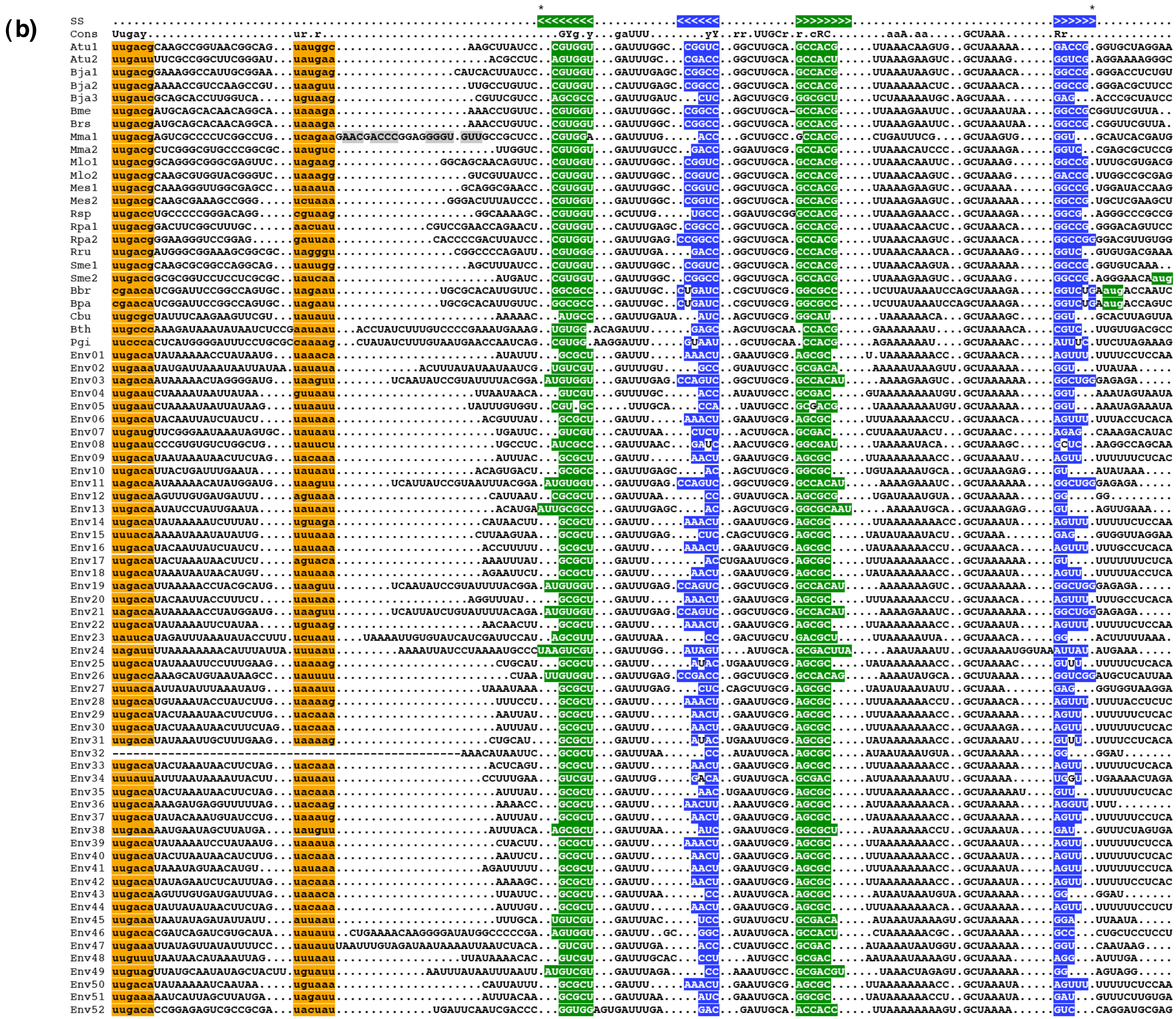

Figure S5 (Page 2/3)

The α-Proteobacterial SAM Riboswitch. **(b)** Sequence Alignment. Details on previous page.

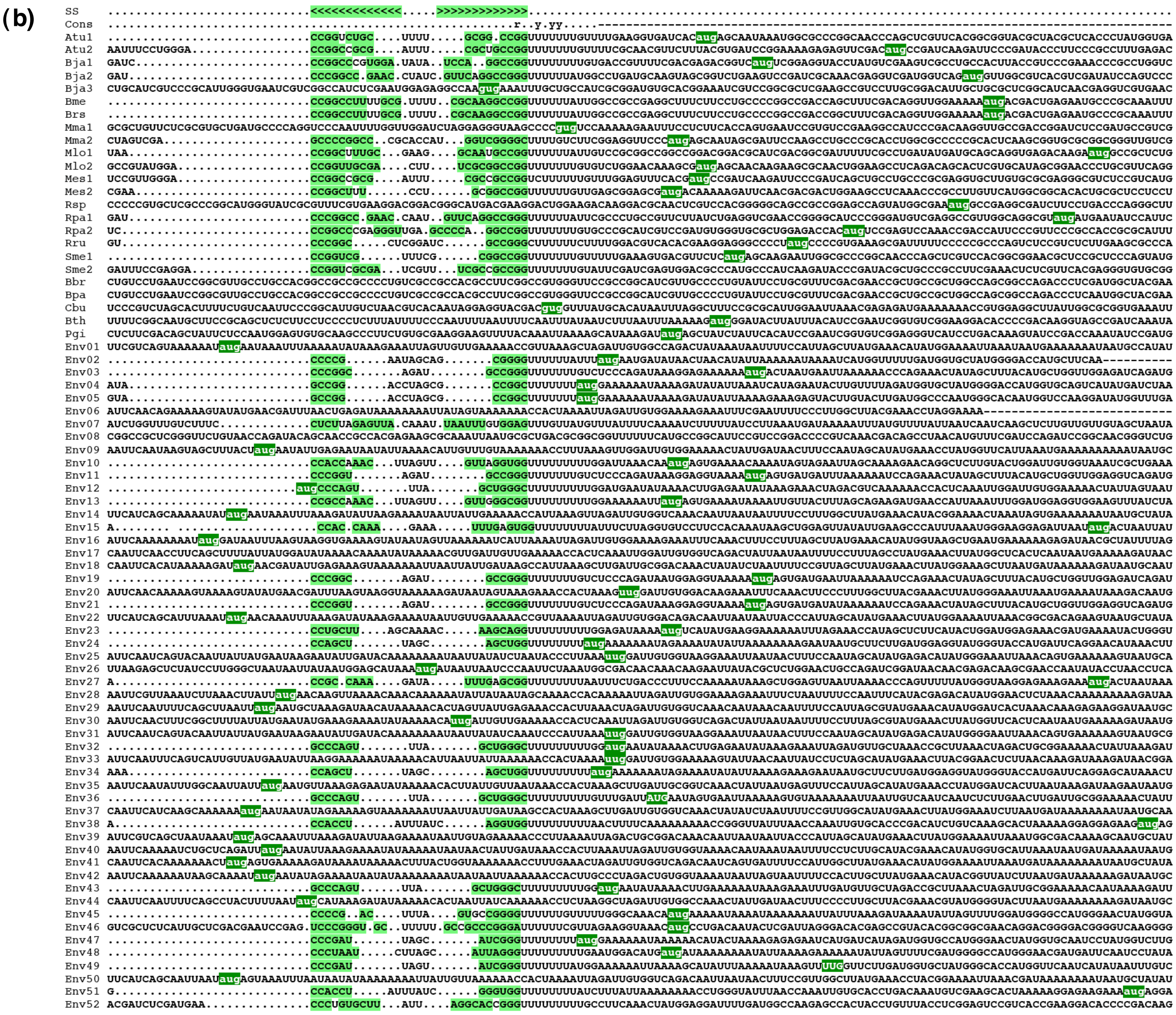

Figure S5 (Page 3/3)

The α-Proteobacterial SAM Riboswitch. **(b)** Sequence Alignment (continued). Details on previous page.
